# Clinical effectiveness of the electrodermal orienting reactivity test for evaluating relapse and recurrence risk in patients hospitalized for depression

**DOI:** 10.1186/s12888-021-03088-3

**Published:** 2021-02-10

**Authors:** Marta Litwińska-Bołtuć, Łukasz Święcicki, Armin Spreco, Toomas Timpka

**Affiliations:** 1 Second Clinic of Psychiatry, Institute of Psychiatry and Neurology, Warsaw, Poland; 2 Department of Health, Medicine, and Caring Sciences, Linköping University, Linköping, Sweden

**Keywords:** Electrodermal activity, Electrodermal hyporeactivity, Relapse of depression, Recurrence of depression

## Abstract

**Background:**

Recurrence is a problem for many patients who have episodes of depression. In experimental settings, hyporeactivity in the Electrodermal Orienting Reactivity (EDOR) test has been observed to be more frequent in these patients. The aim of this study was to investigate the clinical value of this test with regard to a prognosis of episode recurrence in patients hospitalized for depression.

**Methods:**

The study was performed using a cohort design at a specialized psychiatric clinic in Warsaw, Poland. The primary endpoint measure was relapse or recurrence of depression. Data on electrodermal reactivity measured by the EDOR test, clinical status, and psychiatric history were collected at the clinic. Relapse and recurrence data were collected by clinical interviews 1 year after the EDOR test. The predictive (adjusting for confounders) and comparative (relative to other predictors) performance of electrodermal hyporeactivity was assessed using simple and multiple binary logistic regression.

**Results:**

The patient sample included 97 patients aged between 20 and 81 years (mean, 51.2 years). Twenty patients (20.6%) were hyporeactive in the EDOR test. The group of hyporeactive patients did not differ significantly from the reactive group with regard to background factors or clinical status on admission. Forty-seven patients (51.6%) had at least one depressive episode during the follow-up period. In the analysis including potential confounders, the likelihood of relapse or recurrence of depression was nearly five times higher among the hyporeactive patients than the reactive patients (odds ratio [OR], 4.7; 95% confidence interval (CI), 1.3–16.2; *p* = 0.015). In the comparative analysis, only hyporeactivity was found to be associated with recurring episodes (OR, 3.3; 95% CI, 1.1–10.2; *p* = 0.036).

**Conclusions:**

Electrodermal hyporeactivity was associated with a higher risk of relapse or recurrence after discharge among patients hospitalized for depression. This finding warrants further clinical investigations that cover different types of depression and account for causal mechanisms.

**Trial registration:**

The study design was registered in the German Clinical Trials Register (DRKS00010082).

## Introduction

Depression is an increasingly prevalent psychiatric disorder that constitutes a significant burden on health care systems worldwide [1]. For many patients who experience depression, episode recurrence is a severe problem that influences family relations and participation in working life [2]. Approximately 40 to 60% of patients with a first-time episode of major depression develop a subsequent episode [3], and the risk increases further with each new episode [4]. This implies that there is a need for knowledge about reliable determinants of a “relapsing-remitting” course of the disorder [5]. Disturbances in the feed-back function of the hypothalamic-pituitary-adrenal (HPA) axis has consistently been implicated as an etiologic factor of depression. Specifically, acute and chronic stress is recognized to affect the integrity of the axis with regards to its capacity of homeostatic regulation [6]. The HPA-axis is functionally associated with the autonomic nervous system (ANS) [7, 8]. and dysregulation of the ANS is a recognized diagnostic characteristic of depression, with symptoms such as loss of energy, weight loss/gain, and sleep disturbances [9]. Quantification of electrodermal activity (EDA) is a method of monitoring ANS dysregulation through the eccrine sudomotor responses to stimuli [10].

### EDA and depression

EDA quantification involves tonic and phasic components. The tonic components are constituted by the base line Skin Conductance Level (SCL) and the rate of phasic, non-stimulus related responses. Studies of EDA have reported lower SCL in depressed patients compared with healthy controls [11, 12], and lower SCL associated with biological features such as retardation [13–15], inhibition [16], weight loss and gastrointestinal symptoms [14]. The phasic component, which is in focus for clinical interpretations of EDA data, consists of electrodermal representations of localized orienting responses (Skin Conductance Responses, SCRs) [17, 18]. In general, depressed patients are characterized by low and flat EDA profiles. However, the literature on the subject is inconsistent and not all the studies have replicated these findings [19]. Furthermore, the technology and methodologies have changed over time, as have the diagnostic criteria (the terms “reactive” and “endogenous” depression are no longer used), and older studies should be interpreted with caution [20].

### The orienting response

The orienting response is a complex of behavioral and somatic reactions to new, neutral stimuli [10]. It has been suggested that the orienting response has an important role in sustaining homeostasis, i.e. mobilizing the organism to properly respond to a changing environment and thereby increasing its chances for survival. When a stimulus estimated as unimportant and nonthreatening is repeated, the reaction gradually decreases in a process called habituation. In this context, depressed patients have been characterized by fast habituation of the SCR to neutral, auditory stimuli [12, 21, 22]. In more recent studies, different kinds of stimulation have been used, such as watching emotional pictures or movies (happy, sad, amusing, neutral, etc.), performing tasks under pressure, and breathing exercises. However, the results of these studies defy rigorous comparison as a result of variations in the experimental conditions, and further research has been recommended [20].

### Study aims

The use of EDA measurements for identification of patients with depression at risk for episode recurrence has not been studied in clinical practice. The Electrodermal Orienting Reactivity (EDOR) test is a structured measurement of EDA based on the localized orienting response. The aim of this study was to investigate the clinical value of this test with regard to the prognosis of episode recurrence. The hypothesis was that the EDOR test can be used among hospitalized patients to predict recurrence after discharge. More specifically, we wanted to answer the following research questions:

- Are patients hospitalized for depression who test positive for electrodermal hyporeactivity according to the EDOR test at higher risk for recurring episodes than those with a negative test?
- How does the EDOR test perform in comparison with other determinants of recurrence risk in patients hospitalized for depression?

## Methods

A cohort study design was used. The study population consisted of patients hospitalized with a diagnosis of depression at the Affective Disorders Department, II Psychiatric Clinic of the Institute of Psychiatry and Neurology in Warsaw. Patient recruitment was done by random convenience sampling. The primary endpoint for the study was occurrence of relapse or recurrence within 1 year after measurement of electrodermal responsivity (approximately 10 months after discharge from the hospital). All methods were carried out in accordance with relevant guidelines and regulations. The study was approved by the local Bioethics Committee at the Institute of Psychiatry and Neurology in Warsaw (20/2013). The study design was registered in the German Clinical Trials Register (DRKS00010082).

### Patients

Recruitment of patients for the study was performed between 5 August 2014 and 3 March 2016. The inclusion criteria were a primary diagnosis of depression (ICD-10 diagnoses F31.3-F31.9, F32.0-F32.3, F32.8, F32.9, F33.0-F33.4, F34.8, F34.9, F38.0-F38.1, F38.8, F39), aged 18 years or more, and informed consent to take part in the research. Patients who had problems understanding the instructions and those with serious hearing problems were excluded. One-hundred and sixty-five patients admitted to the clinic fulfilled the inclusion criteria. Fifty patients were not provided the informed consent procedure during their hospital stay due to limited availability of research staff. All 115 patients invited consented to participate in the study. Ten patients were after having provided their consent excluded due to problems to understand the instruction for the electrodermal responsivity test. The remaining 105 patients constituted the study population.

### Clinical assessments

During the psychiatric examination, detailed information was collected on age at onset of the disorder, duration of the disorder, lifetime number of depressive episodes, lifetime number of psychiatric hospitalizations, duration of the longest depressive episode, lifetime history of psychotic symptoms, and history of suicide attempts. In addition, medical documentation was used (if available). Validated instruments were used to assess the severity of depression and suicidal ideation: the Montgomery-Asberg Depression Rating Score (MADRS) [23] and the Beck Suicide Intent Scale (BSIS) [24]. The study data were not blinded for the clinicians treating the patient.

### The EDOR test

The equipment and software for the EDOR test was provided by Emotra AB (Gothenburg, Sweden). It includes the EDOR box (containing electrodes and sound generator), professional sound-shielded earphones, and a computer connected to the EDOR box by Bluetooth (https://emotra.se/wp-content/uploads/2018/05/EDOR-Test-and-Emotra-Cloud.pdf). During the 15-min test, a series of sounds (1 s, 90 dB, 1 kHz, with shaped onset and termination) are presented to the patient through the earphones at intervals from 20 to 80 s in the same unpredictable pattern for all patients. Changes in skin conductance of applied constant voltage (0.5 V, according to international standards [25]) are measured. The measurement data are sent online to Emotra AB for analysis and binary classification as “reactive” or “hyporeactive.” The criterion for hyporeactivity was a habituation score of 3 or lower (habituation score is the order number of the first stimulus in a sequence of three stimuli that do not evoke an SCR), meaning that there are SCRs to no more than two stimuli within the first five stimuli [12, 16, 26]. The test results are returned online to the test leader within 2–3 h.

### Follow-up

One year after the EDOR test (approximately 10 months after discharge from the hospital), each patient was contacted, and information about depressive episodes in the follow-up period was obtained. The recurring episodes recorded included relapse (return of symptoms to the full syndrome criteria for an episode during partial or full remission but before recovery) and recurrence (understood as a new depressive episode in a person who had previously experienced a depressive episode and achieved recovery) [10, 27, 28]. The diagnosis of a recurring depressive episode was made based on the patient’s self-reports by the first author (MLB) according to the ICD-10 criteria. Family and friends were contacted if the patient was hard to reach, and official registers and medical documentation were used (if available).

### Statistical analyses

Means and standard deviation were used to describe quantitative (continuous) variables. The number and percentage of patients belonging to each group were used to describe qualitative (categorical) variables. Comparisons between hyporeactive and reactive groups were made using the Mann-Whitney U test for quantitative variables and the chi-squared test for qualitative variables.

Associations between hyporeactivity and relapse or recurrence of depression in the follow-up period were assessed using simple (i.e. hyporeactivity being the only predictor) and multiple binary logistic regression; possible confounding factors were taken into consideration in the latter. Patients were not differentiated by the time of re-appearance of a depressive episode. Confounders taken into consideration were sex, duration of disorder (years), lifetime number of depressive episodes, diagnosis of bipolar affective disorder (yes/no), and result from the MADRS scale. Age at the time of the EDOR test and age at the onset of the disorder were not included in the model because of multicollinearity; duration of the disorder was used instead. To investigate if any of these determinants were associated with relapse and recurrence, a multiple model was fitted using backward elimination (Wald) of non-significant variables (i.e., variables with *p* ≥ 0.05 were eliminated stepwise) and simple models were fitted separately for each of the determinants. Odds ratios (OR) including 95% confidence intervals (CI) were obtained. For all analyses, the level of significance was 0.05. All analyses were performed using SPSS Statistics version 26 (IBM, Armonk, NY).

## Results

### Study population

The final patient sample included 97 of the 105 patients examined (59 women and 38 men). The age range was between 20 and 81 years (mean, 51.2 years). Eight patients were excluded from the analysis because of technical problems (EDOR test interrupted or not recorded properly, test result invalid) or because of a change in the diagnosis during hospitalization (to schizophrenia [F20.2], schizoaffective disorder [F25.2], or adjustment disorder [F43.2]). According to the ICD-10 classification, 61 patients in the final sample were diagnosed with bipolar disorder, 21 with recurrent depressive disorder, and 15 with a depressive episode (not specified).

The patients in the test group had other secondary psychiatric diagnoses, such as personality disorders, anxiety disorders, alcohol or other substance abuse in the past (none of the patients had acute withdrawal syndrome). No patients were diagnosed with or suspected to have dementia at the time of EDOR the test.

### Hyporeactivity and clinical status

In the test group (*n* = 97), 20 patients were hyporeactive in the EDOR test and 77 were reactive. The group of hyporeactive patients did not differ significantly from the reactive group considering any of the clinical variables (sex, age at the time of the EDOR test, age at onset of the disorder, duration of the disorder, lifetime number of depressive episodes, the number of psychiatric hospitalizations, the duration of the longest depressive episode, the number of psychiatric hospitalizations, the presence of psychotic symptoms ever, and score for the MADRS scale) (Table 1). The group of hyporeactive patients also did not differ significantly from the reactive group considering the number of suicide attempts before the EDOR test or any of the scores for the clinical scales used (BSIS and CSSRS; Table 1).

**Table 1:** Patient characteristics and reactivity in the EDOR test

Variable		All patients (*n* = 97)	Hyporeactive patients (*n* = 20)	Reactive patients (*n* = 77)	*p* value
Sex	Women, *n* (%)	59 (60.8)	16 (80.0)	43 (55.8)	0.071
	Men, *n* (%)	38 (39.2)	4 (20.0)	34 (44.2)	
Age (years)	Mean (SD)	51.2 (14.7)	51.4 (18.9)	51.1 (13.5)	0.824
Disorder duration (years)	Mean (SD)	13.7 (11.1)	13.8 (12.5)	13.6 (10.7)	0.848
Age at onset of the disorder (years)	Mean (SD)	37.5 (17.0)	37.6 (20.0)	37.5 (16.3)	0.731
Number of depressive episodes in lifetime	Mean (SD)	7.7 (8.9)	7.6 (8.9)	7.8 (8.9)	0.836
Number of psychiatric hospitalizations in lifetime	Mean (SD)	4.4 (5.5)	4.7 (6.0)	4.3 (5.4)	0.794
Longest depressive episode in lifetime (months)	Mean (SD)	9.8 (10.3)	7.3 (6.0)	10.4 (11.1)	0.188
Diagnosis of bipolar disorder	No, *n* (%)	36 (37.1)	4 (20.0)	32 (41.6)	0.118
	Yes, *n* (%)	61 (62.9)	16 (80.0)	45 (58.4)	
Psychotic symptoms ever in lifetime	No, *n* (%)	72 (74.2)	13 (65.0)	59 (76.6)	0.440
	Yes, *n* (%)	25 (25.8)	7 (35.0)	18 (23.4)	
MADRS score	Mean (SD)	16.9 (10.0)	14.7 (12.5)	17.5 (9.2)	0.194
Suicide attempts before the EDOR test	Mean (SD)	1.0 (1.6)	1.4 (2.2)	0.8 (1.5)	0.662
BSIS part 1	Mean (SD)	14.3 (3.2)	15.1 (3.7)	14.0 (3.1)	0.431
BSIS part 2	Mean (SD)	15.9 (3.3)	15.9 (3.2)	16.0 (3.4)	1.000
Depth of depression according to ICD-10	Severe, *n* (%)	46 (53.5)	11 (57.9)	35 (52.2)	0.261
	Mild/moderate, *n* (%)	25 (29.1)	3 (15.8)	22 (32.8)	
	Psychotic, *n* (%)	15 (17.4)	5 (26.3)	10 (14.9)	

*BSIS* Beck Suicide Intent Scale, *EDOR* Electrodermal Orienting Reactivity, *MADRS* Montgomery-Asberg Depression Rating Score, *SD* standard deviation

### Predictive performance of the EDOR test

Six patients were lost to follow-up. Forty-seven (51.6%) of the remaining 91 patients (33 (45.8%) of the reactive and 14 (73.7%) of the hyporeactive patients) had at least one depressive episode during the follow-up period. In the multiple logistic regression analysis, including potential confounders in the model, the likelihood of relapse or recurrence of depression was nearly five times higher among the hyporeactive patients than the reactive patients (OR, 4.7; 95% CI, 1.3–16.2; *p* = 0.015).

### Comparative performance of the EDOR test

The simple logistic regression analysis showed that hyporeactive patients had more than three times higher likelihood of a recurring episode of depression during the follow-up period compared with the reactive patients (OR, 3.3; 95% CI, 1.1–10.2; *p* = 0.036). Sex, duration of disorder, lifetime number of depressive episodes, diagnosis of bipolar affective disorder, and MADRS score were not associated with the primary endpoint measure in the simple model analysis. In the multiple model determined using backward elimination (Wald) of non-significant variables, only hyporeactivity in the EDOR test was found to be associated with recurring episodes, and therefore the outcome was identical to that of the simple model (OR, 3.3; 95% CI, 1.1–10.2; *p* = 0.036).

## Discussion

We aimed to investigate the clinical value of electrodermal hyporeactivity in depressed patients with regard to the prognosis of recurrence and relapse. It was found that the OR for relapse within 10 months after hospital discharge was four times higher among the hyporeactive patients than the reactive patients. We also observed that the EDA measurements in an analysis of a multiple model of predictors remained as the only determinant of relapse and recurrence risk.

### Hyporeactivity in the EDOR test as a predictor of relapse

This study is the first to report that, in routine clinical practice, electrodermal hyporeactivity is an indicator of increased recurrence and relapse risk among patients hospitalized for depression. None of the other clinical variables or scores included in the study was associated with the probability of relapse and recurrence. Several clinical features have previously been suggested to be associated with such risk, but the number of previous depressive episodes is the most consistently reported [5, 29]. Other determinants, such as the age at onset of the depressive episode or sex, have been less regularly reported [5].

Our results may be interpreted to indicate that hyporeactivity in the EDOR test can be used for evaluation of relapse risk independently of other clinical characteristics. According to previous studies, a low level of EDA tends to remain stable over time independently of symptomatology. For instance, EDA measurements have remained stable after successful electroconvulsive therapy [11, 14], during remission when retested after a few weeks [30] and have only increased slowly when tested after 2 years [26]. Thus, a hypothetical explanation of our findings can be that the EDA patterns observed are an expression of a persisting pathophysiologic condition that increases the susceptibility to relapse [13]. However, this interpretation requires confirmation in future clinical studies where analysis of the causal mechanisms leading to relapse and recurrence is included in the study design.

In this study, no association was observed between a history of suicide attempts and electrodermal hyporeactivity. This observation is in accordance with that the causal pathways leading to suicide attempts [31] and to relapse and recurrence in depression [32], respectively, differ with regard to at least some mechanisms. This finding suggests that separate models of causal pathways should be developed and used in future studies of electrodermal hyporeactivity as a diagnostic tool in these patient groups.

### Study limitations

This study has limitations that need to be considered when interpreting the results. Although all patients were in remission on discharge from the study clinic, we acknowledge that the 1-year follow-up period was insufficient for differentiation between relapse and recurrence [33]. The mean duration from onset to remission of mild depressive episodes is 14–17 weeks and 23 weeks for severe episodes [34]. The additional time needed for recovery varies in the literature, ranging from 8 weeks of continuous remission to 4 months [32, 35]. In addition, a large proportion of patients in the study sample had bipolar disorder, which may have influenced the results. At present, there is no evidence that bipolar hyporeactive patients differ from other hyporeactive patients hospitalized for depression with regard to risk of relapse or recurrence. Nevertheless, this topic requires further study.

Moreover, a convenience sampling method was used for patient recruitment and the clinician who performed the follow-up interview was not blinded to the patient’s clinical data or the EDOR test results. An alternative would have been to conduct the follow-up through register analyses, but this was deemed impracticable due to limited access to primary care records. Based on the fact that patients were invited at random among those admitted to the clinic and the ICD criteria for depression were strictly followed for the follow-up diagnosis, we contend that this circumstance is unlikely to have influenced the results of the study. However, bias cannot be excluded, and further research with more rigorous procedures is warranted.

In addition, it is possible that there are determinants of recurring episodes of depression that were not covered by this study. Our aim was to include measures of the relevant determinants, while also considering the overall burden on the patients. The implementation of the study in a standard clinical setting restricted collection of data outside the clinical routine, and thereby some determinants may have been overlooked, e.g., socioeconomic factors. Moreover, co-morbidity and anti-depressive and other medications were not accounted for in the present analyses. Previous research has suggested the EDA is only marginally affected by pharmacologic treatment [26]. Nonetheless, this topic warrants further investigation [20].

## Conclusion

This study showed that the likelihood for relapse within 10 months after hospital discharge was nearly five times higher among hyporeactive patients hospitalized for depression than among the reactive patients. We also observed that EDA measurement remained as the only determinant of relapse in a multiple model of predictors. A large proportion of patients in the present study sample were diagnosed with bipolar disorder, which may have influenced the results. The findings therefore warrant further clinical investigations involving patients diagnosed with different types of depression.

## Data Availability

The datasets analysed during the current study are not publicly available due to patient confidentiality issues considered in the ethics review but are available from the corresponding author on reasonable request.
